# Improved Solar-Driven Photocatalytic Performance of Highly Crystalline Hydrogenated TiO_2_ Nanofibers with Core-Shell Structure

**DOI:** 10.1038/srep40896

**Published:** 2017-01-19

**Authors:** Ming-Chung Wu, Ching-Hsiang Chen, Wei-Kang Huang, Kai-Chi Hsiao, Ting-Han Lin, Shun-Hsiang Chan, Po-Yeh Wu, Chun-Fu Lu, Yin-Hsuan Chang, Tz-Feng Lin, Kai-Hsiang Hsu, Jen-Fu Hsu, Kun-Mu Lee, Jing-Jong Shyue, Krisztián Kordás, Wei-Fang Su

**Affiliations:** 1 Department of Chemical and Materials Engineering, Chang Gung University, Taoyuan 33302, Taiwan; 2 Center for Reliability Sciences & Technologies, Chang Gung University, Taoyuan 33302, Taiwan; 3 Division of Neonatology, Department of Pediatrics, Chang Gung Memorial Hospital, Taoyuan 33305, Taiwan; 4 Sustainable Energy Development Center, National Taiwan University of Science and Technology, Taipei 10607, Taiwan; 5 Department of Materials Science and Engineering, National Taiwan University, Taipei 10617, Taiwan; 6 Department of Chemical and Materials Engineering, National Central University, Taoyuan 32001, Taiwan; 7 Research Center for Applied Science, Academia Sinica, Taipei 11529, Taiwan; 8 Microelectronics and Materials Physics Laboratories, Department of Electrical Engineering, University of Oulu, FI-90570 Oulu, Finland

## Abstract

Hydrogenated titanium dioxide has attracted intensive research interests in pollutant removal applications due to its high photocatalytic activity. Herein, we demonstrate hydrogenated TiO_2_ nanofibers (H:TiO_2_ NFs) with a core-shell structure prepared by the hydrothermal synthesis and subsequent heat treatment in hydrogen flow. H:TiO_2_ NFs has excellent solar light absorption and photogenerated charge formation behavior as confirmed by optical absorbance, photo-Kelvin force probe microscopy and photoinduced charge carrier dynamics analyses. Photodegradation of various organic dyes such as methyl orange, rhodamine 6G and brilliant green is shown to take place with significantly higher rates on our novel catalyst than on pristine TiO_2_ nanofibers and commercial nanoparticle based photocatalytic materials, which is attributed to surface defects (oxygen vacancy and Ti^3+^ interstitial defect) on the hydrogen treated surface. We propose three properties/mechanisms responsible for the enhanced photocatalytic activity, which are: **(1)** improved absorbance allowing for increased exciton generation, **(2)** highly crystalline anatase TiO_2_ that promotes fast charge transport rate, and **(3)** decreased charge recombination caused by the nanoscopic Schottky junctions at the interface of pristine core and hydrogenated shell thus promoting long-life surface charges. The developed H:TiO_2_ NFs can be helpful for future high performance photocatalysts in environmental applications.

Titanium dioxide (TiO_2_) has drawn a broad attention for its applications in the reduction of global atmospheric pollution^1^, water purification^1^,^2^,^3^,^4^, CO_2_ reduction^5^,^6^,^7^,^8^ and photocatalytic hydrogen production^9^,^10^,^11^ in the past few decades. The thermally and chemically stable TiO_2_ has several practical features (inexpensive, easy to process, and “green material”) that make it a reasonably good choice of selection in many applications^12^,^13^,^14^,^15^,^16^,^17^,^18^,^19^,^20^,^21^,^22^.

The discovery of hydrogenated TiO_2_ materials with visible light absorption has initiated significant interest in solar driven applications^23^,^24^,^25^,^26^,^27^,^28^. Sun *et al*. fabricated hydrogenated TiO_2_ nanoparticles and investigated the hydrogen incorporation into facet-defined anatase TiO_2_ nanocrystals under high H_2_ pressure^29^. Chen *et al*. prepared black TiO_2_ nanoparticles by treating pristine TiO_2_ nanoparticles (crystal-white) under 20 bar pure H_2_ atmosphere at 200 °C for 5 days^30^. The authors also demonstrated an approach to enhance solar absorption by introducing disorder in the surface layers of nanoscale TiO_2_ through hydrogenation^31^. The role of hydrogen in producing lattice disorder was presented in anatase TiO_2_ nanoparticles, and the highly localized nature of the mid-gap states results in spatial separation of exciton in hydrogenated TiO_2_ surface. It accounts for its high photocatalytic efficiency as verified by density functional theory^32^,^33^. Moreover, hydrogenated TiO_2_ nanoparticles exhibit the characteristics of low bandgap, which matches well with visible light absorption^34^,^35^,^36^,^37^. Wang *et al*. reported the hydrogen treatment as a simple and effective strategy to improve the performance of photoelectrochemical water splitting using one dimensional hydrogenated TiO_2_ material^32^. In practical applications, one dimensional material titanate materials are typically better than the corresponding nanoparticles. In addition, Liu *et al*. reported a facile synthesis of hydrogenated TiO_2_ nanobelts. It shows an outstanding UV and visible photocatalytic decomposing of methyl orange and water splitting for hydrogen production^38^. An elongated one dimensional material is easier to achieve a percolated electrical network than with zero-dimensional materials. Bundling of one dimensional material contributes to mechanical strength in tangled networks and thus results in macroscopic films^39^,^40^,^41^. Furthermore, the hydrothermal synthesis has opened up new possibilities for large scale production of TiO_2_ nanofibers by simply thermal treatment of the obtained titanate nanofibers in air^42^.

It is noted that hydrogenated TiO_2_ may worsen the photocatalytic activity under simulated solar light as compared to the pristine material. High pressure hydrogenation can be counterproductive to improve the photocatalytic activity of TiO_2_ due to the formation of bulk vacancy defects^29^. However, we suggest that the suitable staggered band alignments between highly-crystalline TiO_2_ and disordered TiO_2_ have the enhanced photocatalytic activity in hydrogenated TiO_2_, as it provides a driving force for the separation of photoexcited electron^43^,^44^. Hence, the hydrogenated process and its parameters play important role in whether the photocatalytic properties of the material improve of degrade.

In the present work, we demonstrate hydrogenated TiO_2_ nanofibers (H:TiO_2_ NFs) having highly crystalline one dimensional anatase TiO_2_ core and highly defective surface with oxygen vacancies and Ti^3+^ interstitial defects obtained by hydrothermal synthesis and subsequent heat treatment in H_2_ of partial pressure in N_2_ gas flow. An optimal calcination condition is proposed to fine tune the photocatalytic activities. The photo-induced charge carrier distribution and carrier dynamics are systematically investigated to understand the role of surface defects. Photo-induced decoloration of various organic dyes under solar light irradiation confirms the correlation between hydrogenation conditions and the photocatalytic activities.

## Results and Discussion

**Figure 1:**
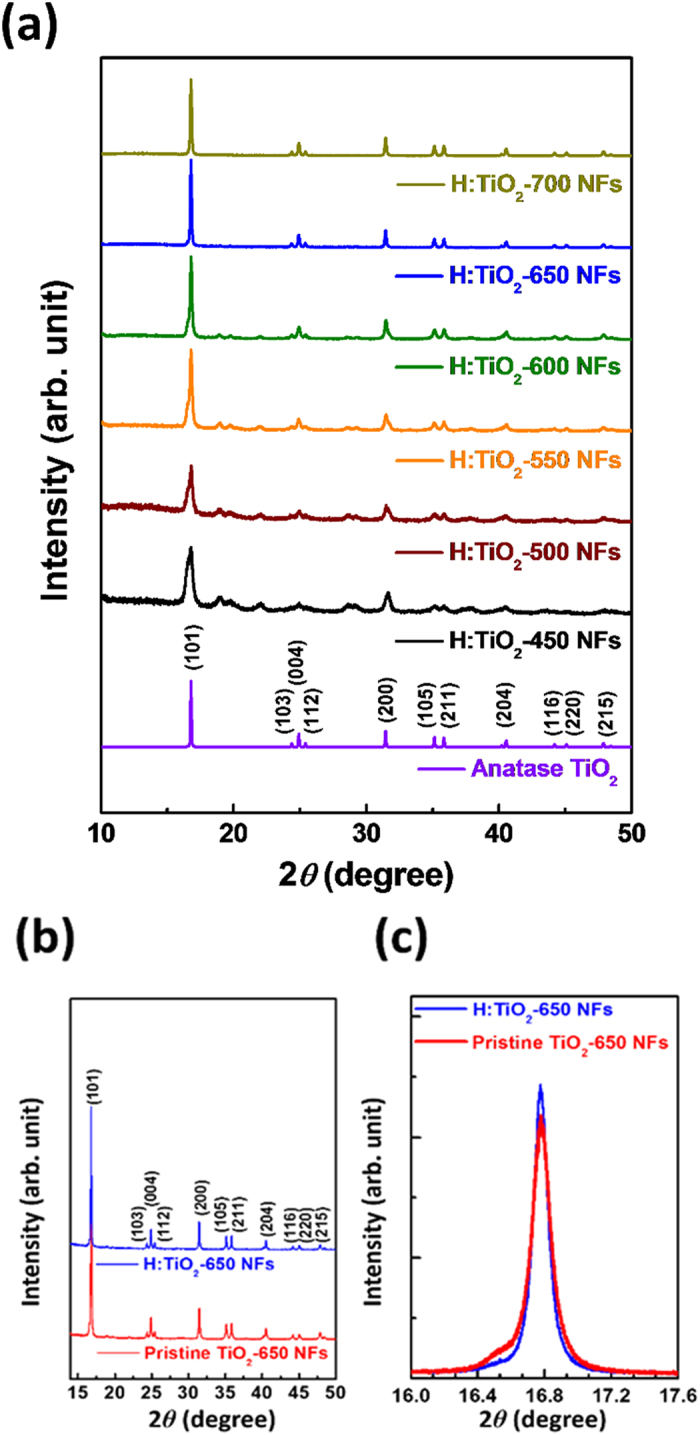
Synchrotron X-ray diffraction of (**a**) various H:TiO_2_-X NFs and the standard powder of anatase TiO_2_ and (**b**) pristine TiO_2_-650 NFs and H:TiO_2_-650 NFs. (**c**) Magnified peak around 16.8°.

The hydrogen sodium titanate nanofibers were calcined at various temperatures in the mixture of H_2_/N_2_ for 12 hrs to find the optimal calcination process that produces the most active photocatalyst. The crystal structure of various H:TiO_2_-X NFs was characterized by synchrotron X-ray diffraction (Fig. 1). (Note, the number in the name of the samples after H:TiO_2_ denotes the calcination temperature.) The results show that H:TiO_2_ NFs calcined below 600 °C comprises a major anatase TiO_2_ phase along with a minor transition phase of monoclinic β-TiO_2_^45^,^46^. The reflection intensity at 2*θ* = 16.8° increases with ascending calcination temperature. The higher calcination temperature improves the ordering of the anatase TiO_2_ lattice. When the applied calcination temperature is above 650 °C, the crystal structure transforms to pure anatase TiO_2_ phase. All diffraction peaks can be perfectly indexed as the body-centred tetragonal structure of anatase TiO_2_, with unit cell parameters a = b = 3.78 Å and c = 9.52 Å [COD ID:720675]. The reflection intensity at 2*θ* = 16.8° decreases at calcination temperatures above 700 °C indicating the formation of rutile TiO_2_ phase from anatase^47^. Also, we synthesized a series of pristine TiO_2_ NFs calcined at various temperatures under the air flow for 12 hrs in comparison with H:TiO_2_ NFs (Fig. 1b). Their mean crystalline domain sizes calculated by Debye-Scherrer equation are 41.6 nm and 51.0 nm, respectively. It says that H:TiO_2_ NFs has larger crystallite size.

**Figure 2:**
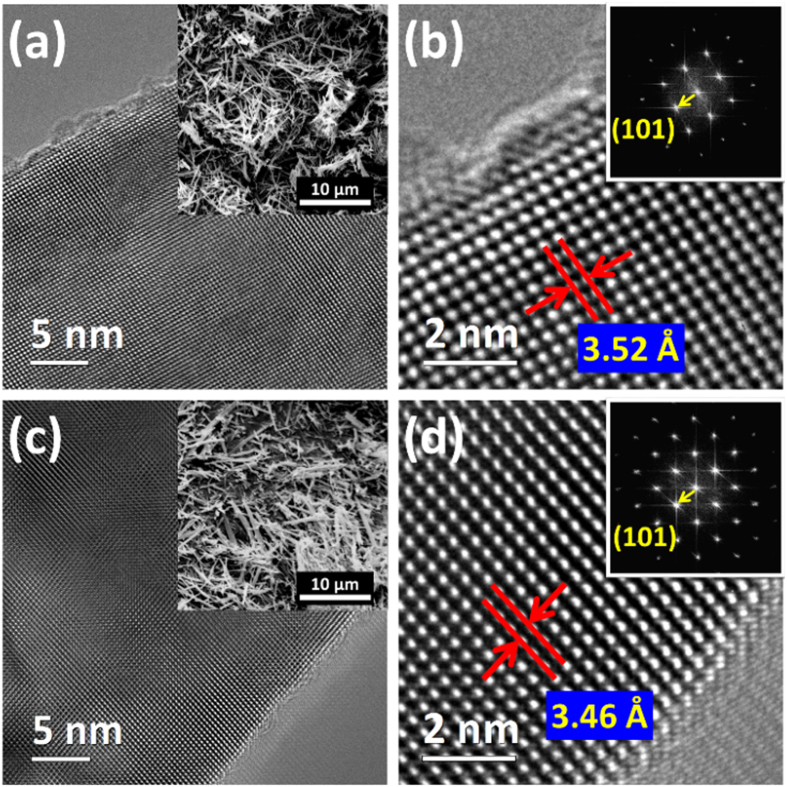
FETEM images and SEM images of (**a**,**b**) pristine TiO_2_ NFs and (**c**,**d**) H:TiO_2_ NFs; the insets of (**b**,**d**) are the corresponding fast Fourier transformed pattern of pristine TiO_2_ NFs and H:TiO_2_ NFs.

The microstructures of pristine TiO_2_-650 NFs and H:TiO_2_-650 NFs are examined by scanning electron microscopy and field emission transmission electron microscopy (Fig. 2). Insets of Fig. 2(a) and (c) show both pristine TiO_2_ NFs and H:TiO_2_ NFs have the length up to several micrometres and diameter of ~100–200 nm. High-magnification lattice images of pristine TiO_2_ NFs and H:TiO_2_ NFs are shown in Fig. 2(b) and (d), respectively. The (101) crystal plane of pristine TiO_2_ NFs and H:TiO_2_ NFs can be observed in the corresponding fast Fourier transformed pattern as displayed in the insets of Fig. 2(b) and (d). The *d* spacing of (101) crystal plane for H:TiO_2_–650 NFs (3.46 Å) is smaller than it for pristine TiO_2_-650 NFs (3.52 Å). The result suggests that hydrogenated process alters the surface lattices on H:TiO_2_ NFs at high calcination temperature. The fine control of the microstructure can warrant extra effort from a materials science point of view.

The chemical compositions of pristine TiO_2_ NFs and H:TiO_2_ NFs were analysed by X-ray photoelectron spectroscopy (XPS) as shown in Table 1. Ti *2p* orbital and O *1s* orbital from pristine TiO_2_ NFs and H:TiO_2_ NFs are shown in Fig. S1 of supplementary information. The XPS results suggest that the crystal surface has oxygen vacancy defects and Ti^3+^ interstitial defects. The resolved Ti *2p* orbital evidences the presence of Ti^3+^ signals at around 457 eV (Fig. S1(a) and (c) of supplementary information), whereas the resolved O *1s* orbital show the existence of O-H bond at around 531.0 eV as shown in Fig. S1(b) and (d) of supplementary information. The oxygen concentration in H:TiO_2_ NFs series is always lower than in pristine TiO_2_ NFs synthesized at the corresponding calcination temperature caused by the lack of oxygen surrounding during calcination carried out under H_2_/N_2_ mixed gas atmosphere. Brunauer-Emmett-Teller (BET) surface area, Barrett-Joyner-Halenda (BJH) cumulative volume of pores and BJH average pore width of pristine TiO_2_-650 NFs and H:TiO_2_-650 NFs were measured by Accelerated Surface Area and Porosimetry System. The detail experimental results is listed in Table 2. The absorption and desorption isotherms and the pore diameter distribution curves of pristine TiO_2_-650 NFs and H:TiO_2_-650 NFs can be found in Fig. S2 of supplementary information. After hydrogenated process, the specific surface area and total pore volume of H:TiO_2_-650 NFs are larger than pristine TiO_2_-650 NFs. The reason could be due to the surface defect formation of H:TiO_2_-650 NFs for nitrogen gas adsorption/desorption, such as oxygen vacancy defects and Ti^3+^ interstitial defects. As a result, the average pore diameter of H:TiO_2_-650 NFs should be decreased after the hydrogenated process due to the formation of small surface defects.

**Figure 3:**
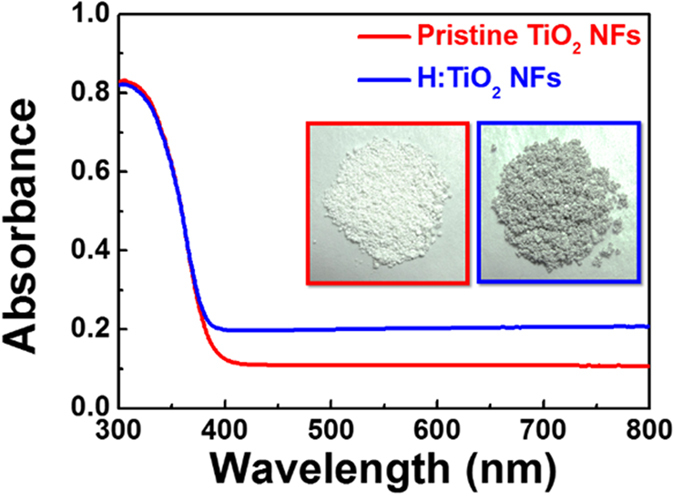
The absorbance spectra of pristine TiO_2_ NFs and H:TiO_2_; the insets are the corresponding powder photos.

Camera images and corresponding absorbance spectrum of pristine TiO_2_ NFs and H:TiO_2_ NFs are shown in the Fig. 3. H:TiO_2_ NFs is having a greyish color with respect to the white pristine TiO_2_ NFs. As compared to pristine TiO_2_ NFs, the absorbance spectrum of H:TiO_2_ NFs is enhanced in the visible region. The bandgaps of pristine TiO_2_ NFs and H:TiO_2_ NFs can be estimated to be approximately 3.17 and 3.14 eV respectively. The enhanced visible absorption behavior could be due to the surface defects, including the oxygen vacancy and the Ti^3+^ interstitial defects. When the Ti^3+^ interstitial defects which reduces Ti^4+^ into Ti^3+^ is on the surface, it introduces mid-gap state into TiO_2_ crystal for enhanced optical absorption^33^,^48^. Computer simulation is used to examine the absorption behavior caused by the oxygen vacancy. All simulations are based on CASTEP (Cambridge Serial Total Energy Package) module in Materials Studio developed by Accelrys Software Inc. Structures of pristine TiO_2_ and H:TiO_2_ with the oxygen vacancy used in this study are made of (3 × 3 × 1) anatase TiO_2_ supercell (Fig. S3 of supplementary information). The theoretical calculations presented in Fig. S4 of supplementary information verify the enhanced absorption of H:TiO_2_ NFs.

**Figure 4:**
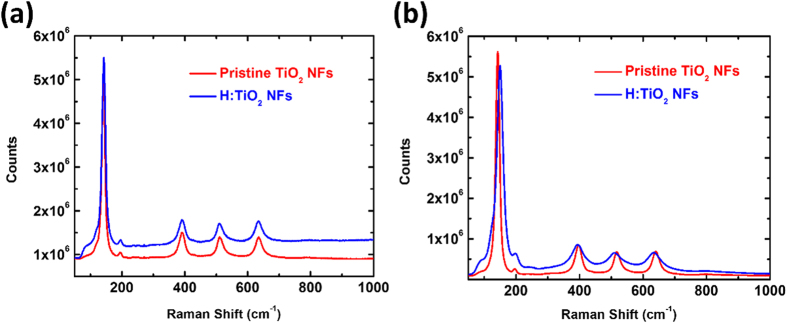
(**a**) 633 nm and (**b**) 532 nm excitation of TERS of pristine TiO_2_-650 NFs and H:TiO_2_-650 NFs.

Tip-enhanced Raman spectroscopy (TERS) gives the information about the surface vibrational modes of the synthesized TiO_2_. Both pristine TiO_2_ NFs and H:TiO_2_ NFs were measured by two-laser TERS system to observe the phase transformation in certain depth profile. The information provided by 532 nm excitation probes more efficiently the outside surface structure, while the 633 nm scatters from the entire volume of the nanowires. As observed, Fig. 4 depicts the inside/outside surface structure of both pristine TiO_2_ NFs and H:TiO_2_ NFs. It is transformed to anatase phase when the calcination temperature was settled at 650 °C. For the anatase TiO_2_ phase, the major Raman bands are located at 144, 200, 398, 515, 517 and 640 cm^−1^, with superimposed Raman bands at 515 and 517 cm^−1^ 49. The individual Raman bands are attributed to the six Raman-active modes of anatase TiO_2_ phase with the symmetries of E_g_, E_g_, B_1g_, A_1g_, B_1g_, and E_g_ (Fig. 4(a)). The outside surface structure of H:TiO_2_ NFs is similar to anatase phase, however, oxygen vacancy defect and the Ti^3+^ interstitial defects (partial TiO_2_ transformed to Ti_2_O_3_) are included. It can be inferred that the formation of Ti^3+^ interstitial defects in anatase results in the red shift of E_g_ phonons (144 and 200 cm^−1^) caused by the multi-phonon B_1g_ of the Ti_2_O_3_. The third E_g_ phonon at 640 cm^−1^ is blue shift affected by the A_1g_ phonon in Ti_2_O_3_. It is also noted that the mixed phase of anatase TiO_2_, oxygen vacancy defect and the Ti^3+^ interstitial defects in the outside surface of H:TiO_2_ NFs has broaden peaks with respect to pristine TiO_2_ NFs as shown in Fig. 4(b)^50^,^51^ TEM microstructure analysis and the TERS reveals that H:TiO_2_ NFs contains a highly crystalline anatase TiO_2_ core and a hydrogenated TiO_2_ shell.

**Figure 5:**
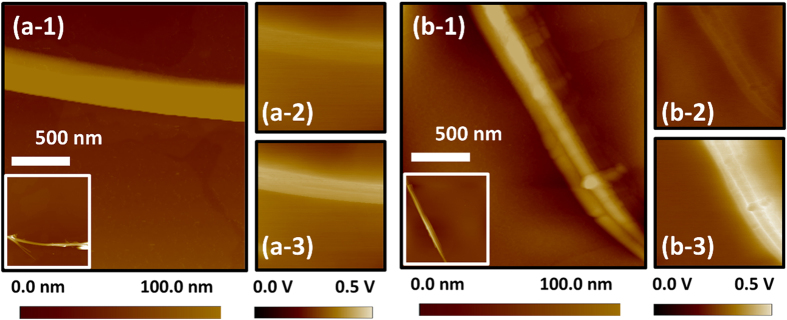
Surface topographic images (**a-1**, **b-1**) and surface potential mappings in the dark (**a-2**, **b-2**) or under UV-B illumination (**a-3**, **b-3**) of single filament of (**a**) pristine TiO_2_ NFs and (b H:TiO_2_ NFs.

The photo-assisted Kelvin probe force microscopy (photo-KPFM) is a useful technique to predict the photocatalytic capability of materials in the development of high performance photocatalysts^52^,^53^. Here, it was applied to elucidate the carrier distribution on pristine TiO_2_ NFs and H:TiO_2_ NFs. Topographic images and surface potential mappings of pristine TiO_2_ NFs and H:TiO_2_ NFs in Fig. 5. Figure 5(a-1) and (b-1) show the topographic images of pristine TiO_2_ NFs and H:TiO_2_ NFs without any ultraviolet light illumination. The average surface potentials of pristine TiO_2_ NFs and H:TiO_2_ NFs are −49.4 mV and −53.4 mV as shown in Fig. 5(a-2) and (b-2), i.e. their surface potential are pretty close. However, the average surface potentials of pristine TiO_2_ NFs and H:TiO_2_ NFs are negative shifted to −118.7 mV and −150.1 mV under ultraviolet light irradiation (i.e., UV-B light with a λ_max_ of 312 nm), respectively. The electron-hole pairs are generated under UV-B irradiation. It results in the splitting of E_*f*_ into quasi-Fermi energy, E_*fn*_ and E_*fp*_, for electrons and holes. E_*fn*_ is usually considered in TiO_2_ because of its characteristic as an n-type semiconductor with electrons being the majority carriers^53^. A considerable drop of surface potential was observed in the case of H:TiO_2_ NFs (−96.7 mV), presenting the larger shift of E_*fn*_ relative to E_*f*_ than that of pristine TiO_2_ NFs (−69.3 mV). The photo-KPFM results show that the H:TiO_2_ NFs has larger photo surface potential shift than pristine TiO_2_ NFs. The accumulated electrons in H:TiO_2_ NFs hence caused the decreased surface potential and shifted E_*fn*_ closer to the TiO_2_ conduction band. It means that the electron-hole pairs can be generated under UV-B light leading to an upward shift of Fermi energy from E_*f*_ to E_*fn*_ and the resulting detected negative shift of surface potential. We assume that the electrons of the H:TiO_2_ NFs excited by pulse laser will be transferred to the H:TiO_2_ NFs surface.

**Figure 6:**
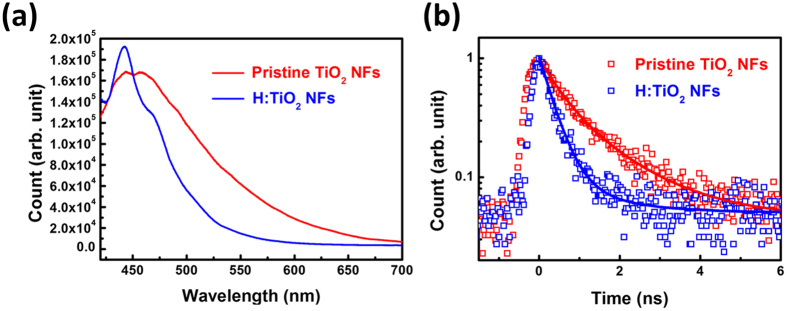
(**a**) PL spectra and (**b**) μ-TRPL spectrum of pristine TiO_2_-650 NFs and H:TiO_2_-650 NFs excited by 375 nm picosecond pulse laser.

Figure 6(a) shows the PL spectra of pristine TiO_2_ NFs and H:TiO_2_ NFs excited by 375 nm picosecond pulsed laser. Intense PL at the position approximately 500 nm from the pristine TiO_2_ NFs is surprising at first glance^54^. Even though pristine TiO_2_ NFs has defect density in the structure so as to give strong PL response around 500 nm, we expected that the H:TiO_2_ NFs would provide higher carrier transport based on the results of the TERS and photo-KPFM in highly crystalline of anatase TiO_2_^55^. In order to address the behaviour of intrinsic PL, the results of micro time-resolved photoluminescence (**μ-**TRPL) was obtained by keeping the wavelength at 425 nm for understanding the carrier transport (Fig. 6(b)). The transient PL decay plots were fitted by bi-exponential kinetics function^56^:


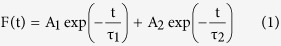


where A_1_ and A_2_ are the corresponding amplitudes. τ_1_ and τ_2_ are fast decay time and slow decay time. The average lifetime was calculated using the following equation^57^:


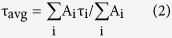


The transient PL decay fitting curve of pristine TiO_2_ NFs and H:TiO_2_ NFs depicts that the hydrogenated process could influence the charge transport efficiency. Table 3 is the summary of the measured fast decay time (τ_1_), slow decay time (τ_2_), and PL average lifetime (τ_avg_) for pristine TiO_2_ NFs and H:TiO_2_ NFs. For the pristine TiO_2_ NFs, the fast decay lifetime is 0.50 ns, the slow decay lifetime is 1.45 ns and their corresponding amplitudes are 54.4% and 45.6% respectively. Surprisingly, the fast decay lifetime of H:TiO_2_ NFs significantly decreases to 0.34 ns and the amplitudes increases to 94.3%. It suggests the improvement of the efficiency of electron transfer to surface and reduces the electron-hole recombination. It is reasonable to know that the average lifetime of the pristine TiO_2_ NFs is 0.93 ns and that of the H:TiO_2_ NFs is 0.40 ns. For the inner structure of the H:TiO_2_ NFs, the highly crystalline anatase TiO_2_ phase could deliver electrons effectively (Fig. 1(c)) It is believed that the outside surface structure of the H:TiO_2_ NFs has large amount of surface defects (including the oxygen vacancy and Ti^3+^ interstitial defect). The hetero-phase junction delivers electron to the surface defect on the outside structure of H:TiO_2_ NFs. The excited electron irradiated by ultraviolet light is located in the surface defects, and it is easily trapped in the mid-state of conduction band which consists with the large negative surface potential at photo-KPFM studies.

**Figure 7:**
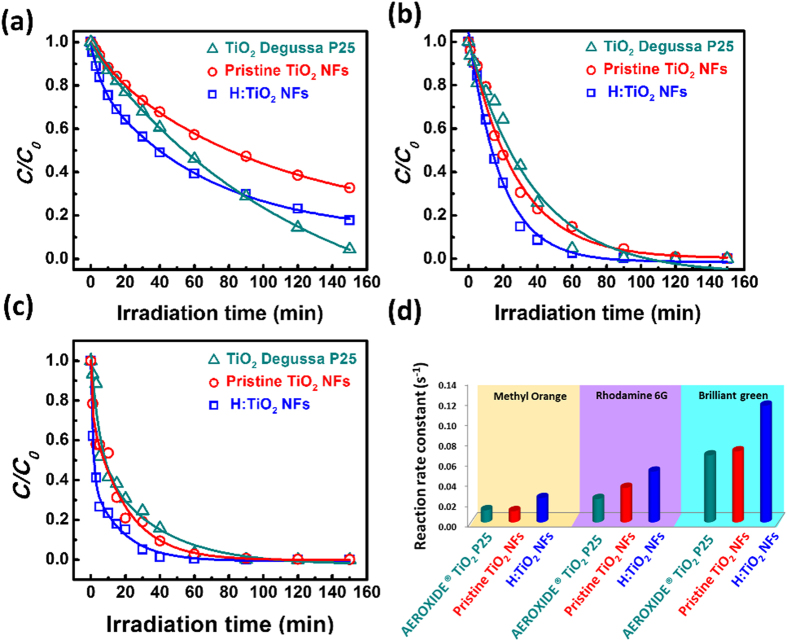
The ***C*****/*****C***_***o***_ curve for the photodegradation of several organic dyes, including (**a**) methyl orange, (**b**) rhodamine 6G, and (**c**) brilliant green, under solar light irradiation using AEROXIDE^®^ TiO_2_ P25, pristine TiO_2_ NFs and H:TiO_2_ NFs. (**d**) The bar charts of photodegradation is reaction rate constants of AEROXIDE^®^ TiO_2_ P25, pristine TiO_2_-650 NFs and H:TiO_2_-650 NFs.

The photodegradation of several organic dyes, including methyl orange, rhodamine 6G and brilliant green, under simulated solar light irradiation were performed by AEROXIDE^®^ TiO_2_ P25, pristine TiO_2_ NFs and H:TiO_2_ NFs. The absorption spectra of methyl orange, rhodamine 6G and brilliant green, as a function of irradiation time were recorded in Fig. 7. The λ_max_ in the measured absorbance spectrum is used to calculate the various organic dye concentration using a calibration curve. The λ_max_ of methyl orange, rhodamine 6G and brilliant green are 464.0, 527.5 and 624.5 nm. The colour of suspension changed from the initial colour to colourless and showed good agreement with first-order kinetics i.e. ***ln**(**C***/***C***_***o***_) = −***kt***; where ***C*** is the concentration of the dye at time ***t**, **C***_***0***_ is the initial concentration, and ***k*** is the apparent reaction rate constant^58^. For the catalyzed photodegradation of various organic dyes, the H:TiO_2_ NFs is superior to pristine TiO_2_ NFs and the commercial AEROXIDE^®^ TiO_2_ P25^59^,^60^. Based on our results thus three mechanisms may be assumed for the high photocatalytic activity of H:TiO_2_ NFs: **(1)** highly crystalline anatase TiO_2_ exhibit the high charge transport rate (Fig. 1), (**2**) the hydrogenated process promotes the visible absorption behaviour to increase exciton generation (Fig. 3), and (**3**) surface charge can photo-induce the electron to decrease charge recombination (Fig. 6). The photocatalytic degradation mechanism of organic dye over H:TiO_2_ NFs is described in equations (3)~(9) 61.


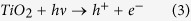



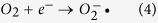



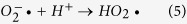



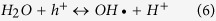



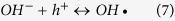



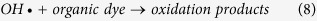



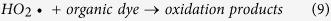


First, when TiO_2_ is irradiated by a light that energy is greater or equal to its bandgap, the photon will excite the valence electron (*e*^−^) to the conduction band and electron-hole pair will be generated. After that, the electron reacts with the oxygen (*O*_2_) to form superoxide ions (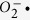). The superoxide ions possess a significant reducing ability, hence it will react with proton (*H*^+^) and reduce to hydroperoxyl radical (*HO*_2_•). Whenever the organic molecules adsorbed on the photocatalyst surface, the hole (*h*^+^) would react with the hydroxide ions (*OH*^−^) or water molecules to form hydroxyl radicals (*OH*•) and *H*^+^. Most of these free radicals behaves excellent oxidation ability, among which *OH*• and *HO*_2_• have the strongest oxidation potential. They will quickly adsorb any organics on the surface of TiO_2_ and undergo oxidation-reduction reactions leading to the production of low molecular weight intermediates. It finally oxidizes these intermediates into environmentally harmless products such as water or carbohydrate. Many studies have focused on the factors that affect the *OH*• formation such as irradiation time, pH and phase structures. Under acidic environment, low pH will promote the formation of *OH*• because of the lower redox potential for hole at valance-band. Also, the phase structures of TiO_2_ affects the formation rate of *OH*• significantly. Amorphous TiO_2_ possesses lots of defect that induces the recombination of electron-hole pairs and suppress the *OH*• formation. Thus, the proper crystalline phase structure design facilitates the photocatalytic phenomenon^62^,^63^. The high-performance photocatalyst should maintain the activities after repeated irradiations. To further evaluate the stability and reusability of the pristine TiO_2_ NFs and H:TiO_2_ NFs, the recycled photocatalytic activities were measured by executing repeated degradation reaction of methyl orange over pristine TiO_2_ NFs and H:TiO_2_ NFs for five recycling runs under UV-B light irradiation. The photostability testing of pristine TiO_2_ NFs and H:TiO_2_ NFs were shown in Fig. S5 of supplementary information. H:TiO_2_ NFs exhibits the higher stability behavior than pristine TiO_2_ NFs under UV-B irradiation. After two recycling runs, the photostability testing for H:TiO_2_-650 NFs shows no obvious decay. Hence, H:TiO_2_ NFs is more chemically stable than pristine TiO_2_ NFs, and it cannot be easily photocorroded under UV light irradiation.

**Figure 8:**
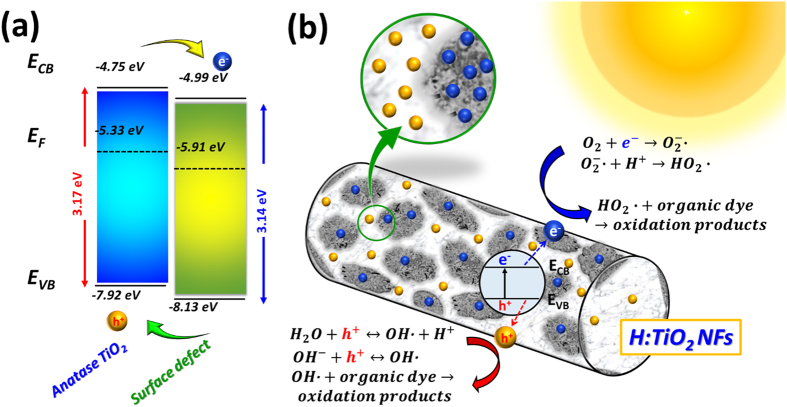
(**a**) The schematic diagram of the possible band alignment between the surface defect (oxygen vacancy and Ti^3+^ interstitial defect) and anatase TiO_2_. (**b**) Illustration of the mechanism of photocatalytic degradation of organic dye over H:TiO_2_ NFs.

When H:TiO_2_ NFs absorbs solar light with energy larger than its bandgap, excitons are generated. The electrons generated in H:TiO_2_ NFs are effectively transferred to the surface defect, and it can capture the photogenerated electrons effectively thus reducing the rate of electron-hole recombination. In order to confirm the position of valence band and conduction band, the pristine TiO_2_ NFs and H:TiO_2_ NFs were measured by Ultraviolet Photoelectron Spectroscopy (UPS). The UPS spectra of pristine TiO_2_ NFs and H:TiO_2_ NFs are shown in Fig. S6(a) of the supplementary information. Work function (WF) is derived from subtracting the cut-off binding energy with the photon energy (21.22 eV). The WF of pristine TiO_2_ NFs and H:TiO_2_ NFs are 5.33 and 5.91 eV, respectively^64^. The expanded valence spectra of pristine TiO_2_ NFs and H:TiO_2_ NFs are shown in Fig. S6(b) of the supplementary information. The valence band maximum (VBM) of pristine TiO_2_ NFs and H:TiO_2_ NFs are found to be located at about 2.59 and 2.22 eV below the Fermi level (*E*_*f*_). Hence, the VBM position of the pristine TiO_2_ NFs and H:TiO_2_ NFs are −7.92 and −8.13 eV, respectively. The schematic diagram of the band alignment between the surface defect and anatase TiO_2_ are shown in Fig. 8(a). The holes generated in H:TiO_2_ NFs could stay on the area without surface defects due to the VBM of H:TiO_2_ NFs is lower than that of pristine TiO_2_ NFs. If the holes are not directly recombined with electrons in H:TiO_2_ NFs, they are able to be further transferred to react with the organic dyes. During the charge separation and migration processes, some of the excited charges may recombine. If the electrons generated in H:TiO_2_ NFs are effectively transferred to the oxygen vacancy and Ti^3+^ interstitial defects, it captures the photogenerated electrons effectively and to reduce the rate of electron-hole recombination. In addition, the electronic structure and optical properties were also calculated to confirm the result with UPS study by the first-principles calculations based on density functional theory. The density of states of pristine TiO_2_ and H:TiO_2_ is also calculated, and the detail data of structure is shown in Fig. S7 of the supplementary information. Each electronic structure was analyzed in order to obtain the origin of the band discontinuity. In comparison of density of state, the conduction band and valence band of both the pristine TiO_2_ and H:TiO_2_ is attributed to the Ti *3d* and O *2p* orbital, respectively. The results show that a mid-state of H:TiO_2_ can be considered as an extension of conduction band. As a consequence, the mid-state of H:TiO_2_ could narrow the band gap and lead to the excited electrons richly transported from conduction band to new mid-state (Fig. S7 of the supplementary information). Figure 8(b) illustrates the outside material of H:TiO_2_ NFs is consisted of the surface defects, including the oxygen vacancy and Ti^3+^ interstitial, and highly crystalline anatase TiO_2_. The photocatalytic activity depends on the amount of working electrons and holes on the surface of the photocatalyst. The H:TiO_2_ NFs with a core-shell structure prepared by the hydrothermal synthesis and subsequent heat treatment at low H_2_ partial pressure in the N_2_ gas flow can be helpful for searching the high-performance visible-light-active photocatalyst in the field of degradation of pollutants with solar light.

## Conclusion

In summary, H:TiO_2_ NFs was prepared by a safe and easy process, and its characteristics were studied to understand the correlation between the hydrogenated process and the solar-light-assisted photocatalytic performance. The high absorption in solar light is due to the oxygen vacancy and Ti^3+^ interstitial defects on the surface of the H:TiO_2_ NFs. The photo-KPFM analysis and μ-TRPL confirms the lower recombination rate and higher charge transport in H:TiO_2_ NFs compared with pristine TiO_2_ NFs. For the photodegradation of various organic dyes, including methyl orange, rhodamine 6G and brilliant green, H:TiO_2_ NFs gave the fastest decoloration phenomenon under solar light irradiation than TiO_2_ P25 and pristine TiO_2_ NFs. Our study indicates that the significant photodegradation activity is obtained by adding the surface defect (the oxygen vacancy and Ti^3+^ interstitial defect) into TiO_2_ NFs surface. Three mechanisms were elucidated: **(1)** enhancement in absorbance to increase exciton generation, **(2)** highly crystalline anatase TiO_2_ to increase the charge transport rate, and **(3)** decreased charge recombination to increase surface charge. The result illustrates a soft controlling of the hetero-phase junction and highly crystalline anatase TiO_2_. It may strongly change the ability of the materials in photodegradation of pollutants.

## Methods

### Preparation of H:TiO_2_ NFs

For the preparation of H:TiO_2_ NFs, we suspend 2.50 g TiO_2_ anatase powder (Aldrich, 98%) in 62.5 mL of 10.0 M NaOH aqueous solution, followed by a treatment in a Teflon-lined autoclave at 150 °C for 24 hrs, applying revolving around its short axis. Then, sodium titanate NFs was then washed in 0.10 M HCl to exchange sodium ions for protons. The neutralized product was washed with deionized water and finally filtered and dried in the air at 70 °C to obtain the hydrogen sodium titanate NFs. The hydrogen sodium titanate NFs were calcined at in 15% H_2_ (in N_2_ buffer) flow for 12 hrs to obtain the various H:TiO_2_-xxx NFs.

### Characterization of TiO_2_ NFs

The crystal structure of pristine TiO_2_-650 NFs and H:TiO_2_-650 NFs were determined by synchrotron X-ray spectroscopy (l~1.025 Å) on beam line 13A1 of the National Synchrotron Radiation Research Cen*t*er (NSRRC) in Taiwan. Spherical-aberration corrected field emission transmission electron microscope (JEOL, JEM-ARM200FTH, Japan) was used to observe the microstructures of pristine TiO_2_-650 NFs and H:TiO_2_-650 NFs. In addition, UV-vis absorption spectra of various synthesized TiO_2_ samples were measured by absorption spectrophotometer (Jasco Analytical Instruments, V-650, Japan) in the 200–800 nm wavelength range. XPS (X-ray photoelectron spectroscopy) spectra were recorded with a PHI 5000 Versa Probe system (ULVAC-PHI, Chigasaki) using a micro focused (100 μm, 25 W) Al X-ray beam. BET surface area, BJH cumulative volume of pores and BJH average pore width of pristine TiO_2_-650 NFs and H:TiO_2_-650 NFs were measured by Accelerated Surface Area and Porosimetry System (ASAP 2020, Micromeritics). The system of micro time-resolved photoluminescence (μ-TRPL) with one lasers as Picosecond diode laser driver with 375 nm Laser head (with integrated collimator and TE cooler for temperature stabilization) was integrated by UniNanoTech Co., Ltd. Andor iDus CCD with 1024 × 128 pixels was used to take the PL signal and the Pico Quant PMT Detector head with 200–820 nm, <250 ps IRF was integrated to take the μ-TRPL signal. In a particular measurement, tip-enhanced Raman spectroscopy was performed using a UniRAM system (UniNanoTech) combined with MV4000 (Nanonics) scanning probe stage at excitation wavelengths of 532 nm (10 mW) and 633 nm (13 mW). The signal collection was detected by a CCD panel having 1024 × 256 pixels. The work function and HOMO (Highest Occupied Molecular Orbital) of pristine TiO_2_-650 NFs and H:TiO_2_-650 NFs were measured by ultraviolet photoelectron spectroscopy (UPS, ULVAC-PHI, Chigasaki) using ultraviolet light source of He I emission (21.2 eV, B50 W) and take-off angle of 90°. Low energy secondary electrons were collected by applying 10 V dc to specimens.

### Photo-KPFM analysis

The surface potential mapping was measured using a photo-KPFM (Kelvin probe force microscope, Digital Instruments, Nanoscopes III). Pristine TiO_2_-650 NFs and H:TiO_2_-650 NFs were dispersed in ethanol and spin-coated on a gold coated (thickness of 100 nm) silicon wafer and then dried before analyses. The experimental setup of photo-KPFM was conducted using UV-B light (λ_max_ ~ 312 nm, 8 W) exposure. The surface potential maps of samples were taken with and without illumination at room temperature. N-type silicon cantilever (Nanosensors, average force constant of 2.8 N/m) is coated with chromium as a buffering layer. A platinum-iridium5 alloy was used as a conductive layer. With this method, the height variation and contact potential by electrostatic force between the conductive tip and the surface of the samples are measured simultaneously. A line is scanned using AFM in tapping mode to acquire the topographic information of the material, then the same line is rescanned with the tip lifted to a height of 20 nm. During the second scan, V_DC_ is applied at the tip to nullify the electrostatic oscillations, position by position, and the contact potential difference is observed and measured. The surface potential distributions of pristine TiO_2_-650 NFs and H:TiO_2_-650 NFs were mapped in the dark or under the illumination of a UV-B lamp (Sankyo Denki, G8T5E, 8 W). In addition, the function of the cross-section analysis was used to get detailed information on the topographic height and the surface potential across the selected line. The surface potential was obtained in the dark or under UV-B illumination. The surface potential difference is then denoted as the photo surface potential shift.

### Photodegradation of organic dyes under solar simulator

AEROXIDE^®^ TiO_2_ P25, pristine TiO_2_-650 NFs and H:TiO_2_-650 NFs were tested in the photodegradation of organic dyes, including methyl orange (C_14_H_14_N_3_NaO_3_S, Acros Organics, pure), rhodamine 6G (C_28_H_31_N_3_ClN_2_O_3_, Acros Organics, pure), and brilliant green (C_27_H_34_N_3_N_2_O_4_S, Acros Organics, pure) under solar light irradiation. AM 1.5G solar simulator (Yamashita Denso, YSS-180S) was used as the irradiation source for the photodegradation of various organic dyes. The intensity of the simulated sunlight was calibrated to be 100 mW/cm^2^ by a silicon photodiode. In this experiment, 20.0 mg of AEROXIDE^®^ TiO_2_ P25, pristine TiO_2_-650 NFs and H:TiO_2_-650 NFs were sonicated for 10 min in 150 mL of 10.0 ppm organic dye aqueous solution, respectively. The temperature of the stirred dispersion was kept near room temperature. The distance between each lamp and reactor was about 15.0 cm. Before the actual photodegradation experiments, the suspensions were left to relax for 30 min in order to minimize the error of the dye concentration measurements caused by initial surface adsorption. After centrifuging for 15 min at 5000 rpm, the absorption spectrum of the retained organic dye and its derivatives in the supernatant was recorded by absorption spectrophotometer (JASCO Analytical Instruments, V-630, Japan) in the 300–900 nm wavelength range.

### Photostability Testing for pristine TiO_2_-650 NFs and H:TiO_2_-650 NFs

A photostability testing on the pristine TiO_2_-650 NFs and H:TiO_2_-650 NFs was carried out according to the literature^21^. In this measurement of photostability testing, 20.0 mg of pristine TiO_2_-650 NFs and H:TiO_2_-650 NFs were sonicated for 10 min in 300 mL of 10.0 ppm methyl orange aqueous solution, respectively. The temperature of the stirred dispersion was kept near room temperature. The distance between the 4 pieces of UV-B lamp (Sankyo Denki, G8T5E, 8 W)) and reactor was about 10.0 cm. After the reaction of first run testing under UV-B light irradiation, the suspensions were centrifuged to obtain the photocatalyst, which was washed with ethanol and deionized water carefully and then dried at 105 °C for 24 hr. The fresh 10.0 ppm methyl orange aqueous solution was mixed with the used photocatalyst to perform the 2^nd^ run photoactivity testing. Similarly, the recycled 3^rd^, 4^th^ and 5^th^ tests were also performed.

### Computational simulation

Computational simulation used in this paper is based on density functional theory with a GGA-PBE (Generalized Gradient Approximation Perdew-Burke-Ernzerhof) functional implemented in CASTEP which uses a plane wave basis set to expand the election wave function. As for the pseudopotential, two setups are adopted depending on the characteristic we are simulating. This is because that pseudopotential will have better accuracy in predicting the properties they suit. In the simulations of absorption spectrum and density of states (DOS), TiO_2_ is modelled by a (3 × 3 × 1) supercell with/without oxygen vacancy (Fig. S2 of supplementary information). In this case, a norm-conserving pseudopotential is used due to its accuracy in predicting optical properties, and the calculations are conducted with an energy cutoff of 450.0 eV and a k-point set of 1 × 1 × 2.

## Additional Information

**How to cite this article**: Wu, M.-C. *et al*. Improved Solar-Driven Photocatalytic Performance of Highly Crystalline Hydrogenated TiO_2_ Nanofibers with Core-Shell Structure. *Sci. Rep.* **7**, 40896; doi: 10.1038/srep40896 (2017).

**Publisher's note:** Springer Nature remains neutral with regard to jurisdictional claims in published maps and institutional affiliations.

## Supplementary Material

Supplementary Information

## Figures and Tables

**Table 1:** The ratios of Ti^3+^/Ti and O/Ti in pristine TiO_2_ NFs and H:TiO_2_ NFs.

Sample	Ti^3+^/Ti (%)	O/Ti (%)
Pristine TiO_2_ NFs	13.56	164.13
H:TiO_2_ NFs	18.14	158.42

**Table 2:** Specific surface area (**S**_**BET**_), total pore volume and average pore size of the prepared pristine TiO_2_-650 NFs and H:TiO_2_-650 NFs.

Sample	S_BET_ (m^2^/g)	Pore Volume (cm^3^/g)		Pore Diameter (nm)	
		BJH Adsorption	BJH Desorption	BJH Adsorption	BJH Desorption
Pristine TiO_2_ NFs	27.12	0.105	0.105	14.86	14.14
H:TiO_2_ NFs	32.91	0.126	0.126	14.50	13.82

**Table 3:** Summary of the measured fast decay time (τ_1_), slow decay time (τ_2_), and PL average lifetime (τ_avg_) for pristine TiO_2_ NFs and H:TiO_2_ NFs.

Sample	A_1_ (%)	τ_1_ (ns)	A_2_ (%)	τ_2_ (ns)	τ_avg_ (ns)
Pristine TiO_2_ NFs	54.4	0.50	45.6	1.45	0.93
H:TiO_2_ NFs	94.3	0.34	5.7	1.32	0.40
