# Supplementary material for: Improved Solar-Driven Photocatalytic Performance of Highly Crystalline Hydrogenated TiO2 Nanofibers with Core-Shell Structure
Source: Sci Rep. 2017 Jan 19;7:40896. doi: 10.1038/srep40896 (PMC5244370; doi:10.1038/srep40896)
Supplement: Supplementary Information [file srep40896-s1.pdf]

# Improved Solar-Driven Photocatalytic Performance of Highly Crystalline Hydrogenated $TiO_2$ Nanofibers with Core-Shell Structure

Ming-Chung Wu[1,2,3*], Ching-Hsiang Chen[4], Wei-Kang Huang[1], Kai-Chi Hsiao[1], Ting-Han Lin[1], Shun-Hsiang Chan[1], Po-Yeh Wu[1], Chun-Fu Lu[5], Yin-Hsuan Chang[1], Tz-Feng Lin[1], Kai-Hsiang Hsu[3], Jen-Fu Hsu[3], Kun-Mu Lee[6], Jing-Jong Shyue[5,7], Krisztián Kordás[8] & Wei-Fang Su[5]

1. Department of Chemical and Materials Engineering, Chang Gung University, Taoyuan 33302, Taiwan
   E-mail: mingchungwu@mail.cgu.edu.tw; Fax:+886-3-2118668; Tel: +886-3-2118800ext.3545
2. Center for Reliability Sciences & Technologies, Chang Gung University, Taoyuan 33302, Taiwan
3. Division of Neonatology, Department of Pediatrics, Chang Gung Memorial Hospital, Taoyuan 33305, Taiwan
4. Sustainable Energy Development Center, National Taiwan University of Science and Technology, Taipei 10607, Taiwan
5. Department of Materials Science and Engineering, National Taiwan University, Taipei 10617, Taiwan
6. Department of Chemical and Materials Engineering, National Central University, Taoyuan 32001, Taiwan
7. Research Center for Applied Science, Academia Sinica, Taipei 11529, Taiwan
8. Microelectronics and Materials Physics Laboratories, Department of Electrical Engineering, University of Oulu, FI-90570 Oulu, Finland

**Figure S1**

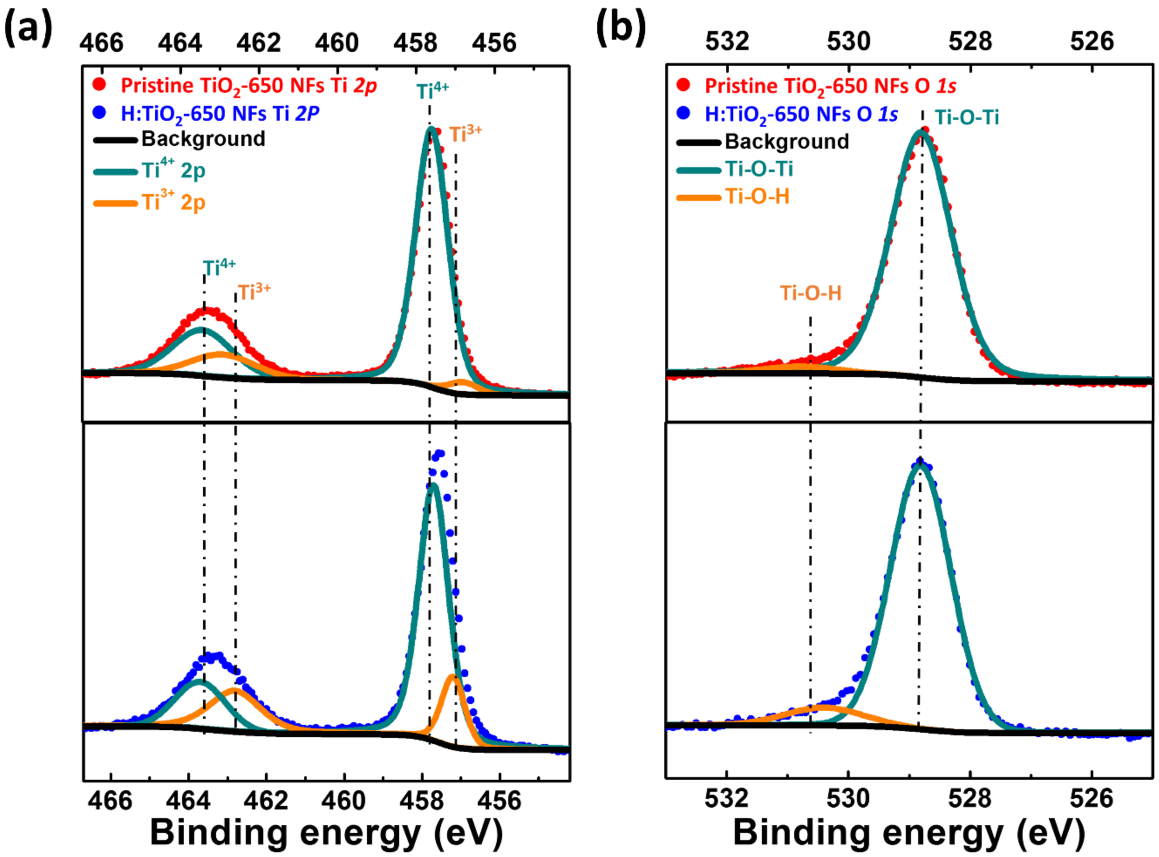


**Figure S1.** X-ray photoelectron spectra of **(a)** Ti *2p* orbital and **(b)** O *1s* orbital of pristine $TiO_2$-650 NFs and H:$TiO_2$-650 NFs. Red spot is O 1s XPS spectrum of pristine $TiO_2$-650 NFs, blue spot is O *1s* XPS of H:$TiO_2$-650 NFs, green line is Ti-O-Ti fitting line and orange line is Ti-O-H fitting line.

**Figure S2**

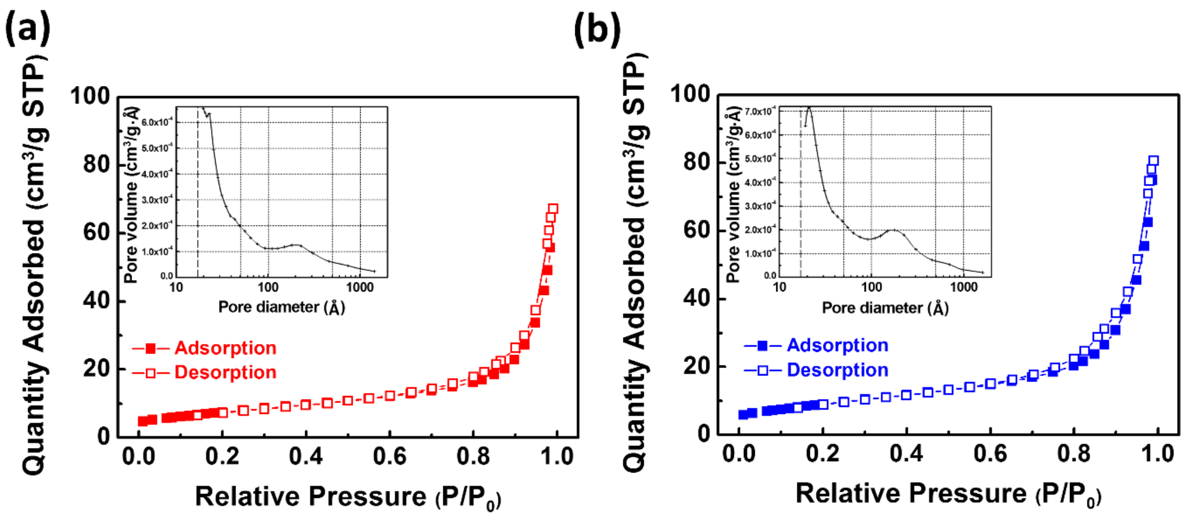


**Figure S2**. The absorption and desorption isotherms of **(a)** pristine $TiO_2$-650 NFs and **(b)** H:$TiO_2$-650 NFs. The inset is the pore diameter distribution curve.

**Figure S3**

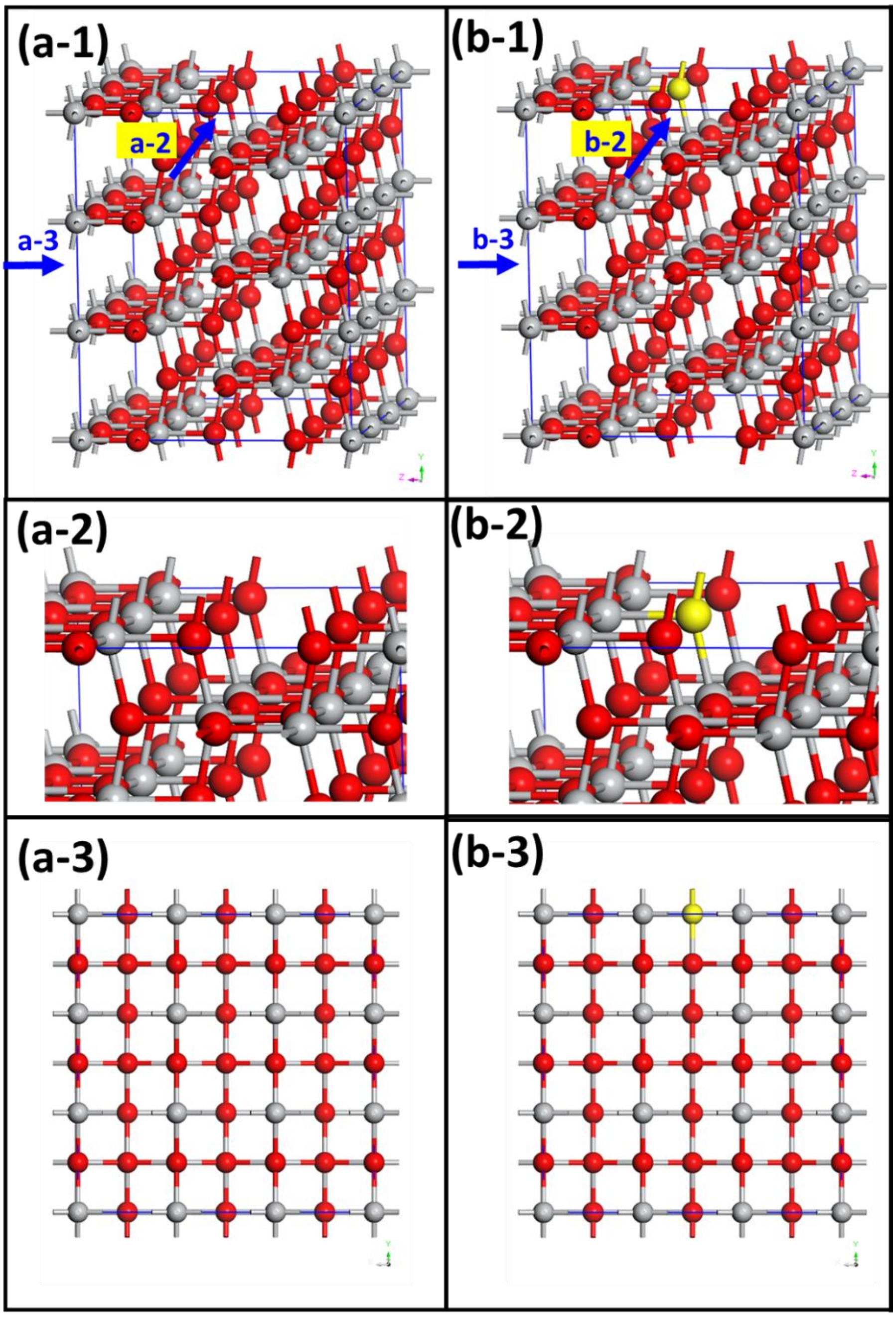


**Figure S3.** Atomic structures of **(a-1)** pristine $TiO_2$ and **(b-1)** H:$TiO_2$ are demonstrated; all models are made of (3×3×1) anatase $TiO_2$ supercell. **(a-2, b-2)** and **(a-3, b-3)** are images observed from relevant viewpoints. The red ball represents the oxygen atom, the gray ball represents the titanium atom, and the yellow mark represents the position of an oxygen vacancy.

**Figure S4**

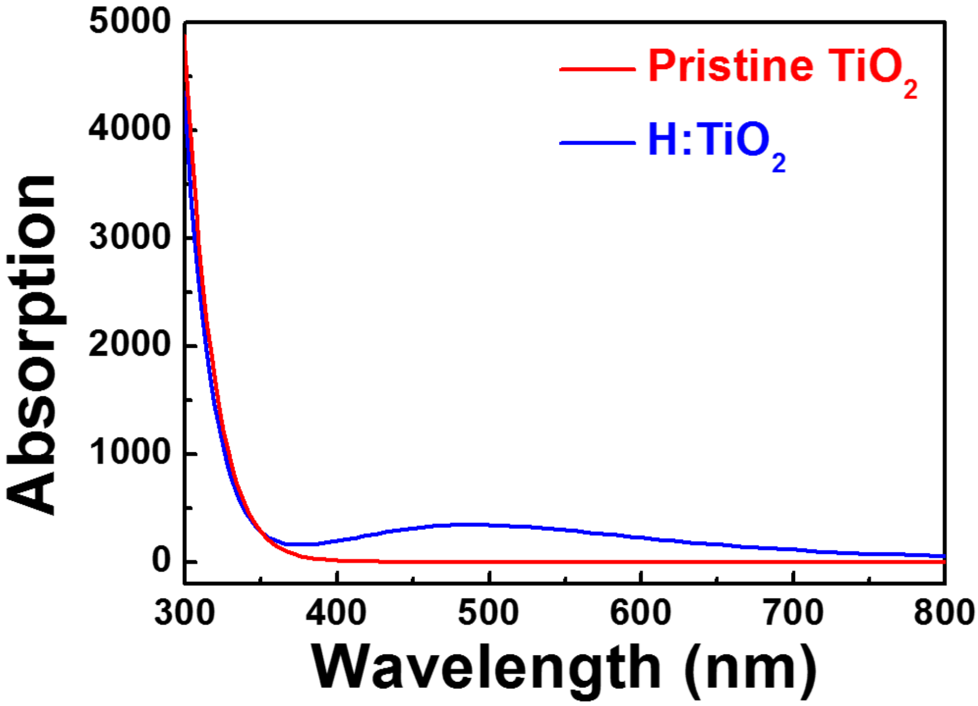


**Figure S4.** Theoretical absorption spectra of pristine $TiO_2$ and H:$TiO_2$. A set of Hubbard U parameter (5.5 eV for 3*d* electrons of Ti and 2.5 eV for 2*p* electrons of O) is used to fix an underestimated band gap of $TiO_2$. Increased visible absorption can be observed in H:$TiO_2$ NFs compared with pristine $TiO_2$ NFs.

**Figure S5**

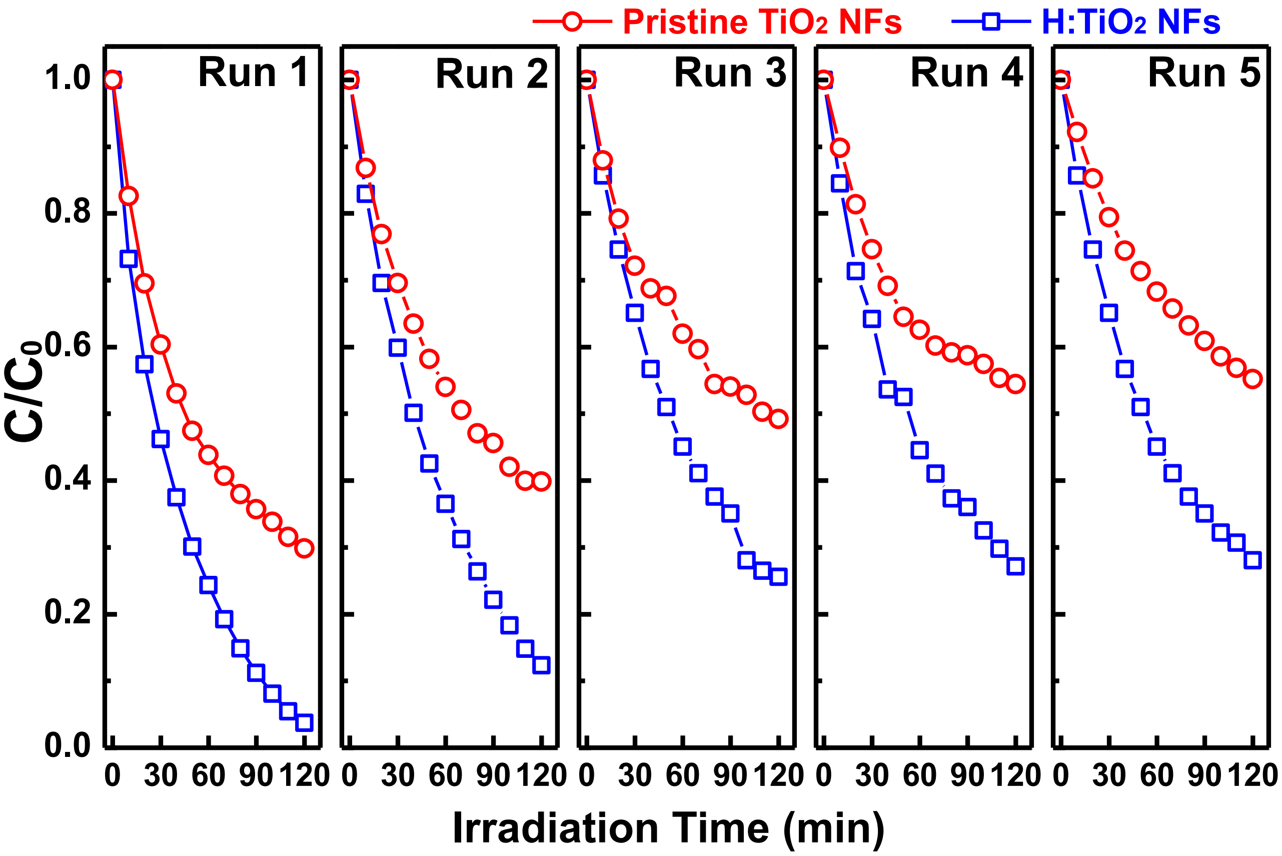


**Figure S5.** Stability tests for the photodegradation of methyl orange over pristine $TiO_2$-650 NFs and H:$TiO_2$-650 NFs under UV-B light irradiation.

**Figure S6**

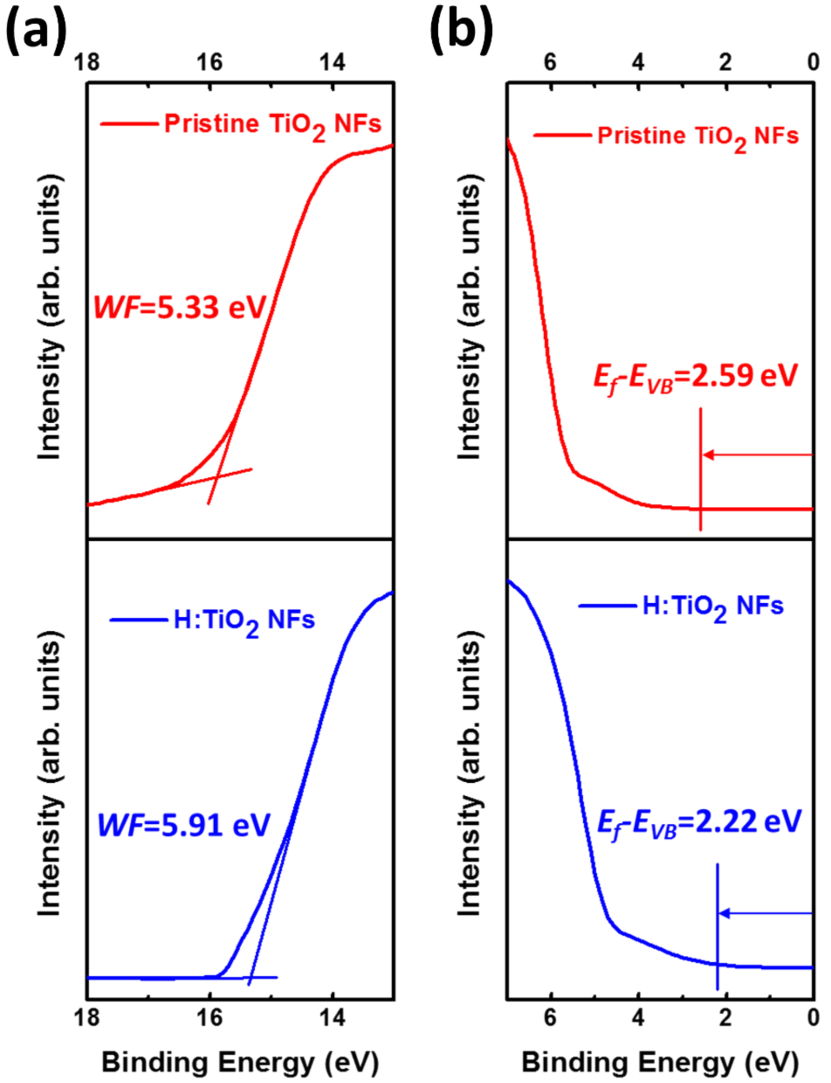


**Figure S6.** UPS spectra of pristine $TiO_2$-650 NFs and H:$TiO_2$-650 NFs. **(a)** secondary-electron cut-off, and **(b)** the valence-band region.

**Figure S7**

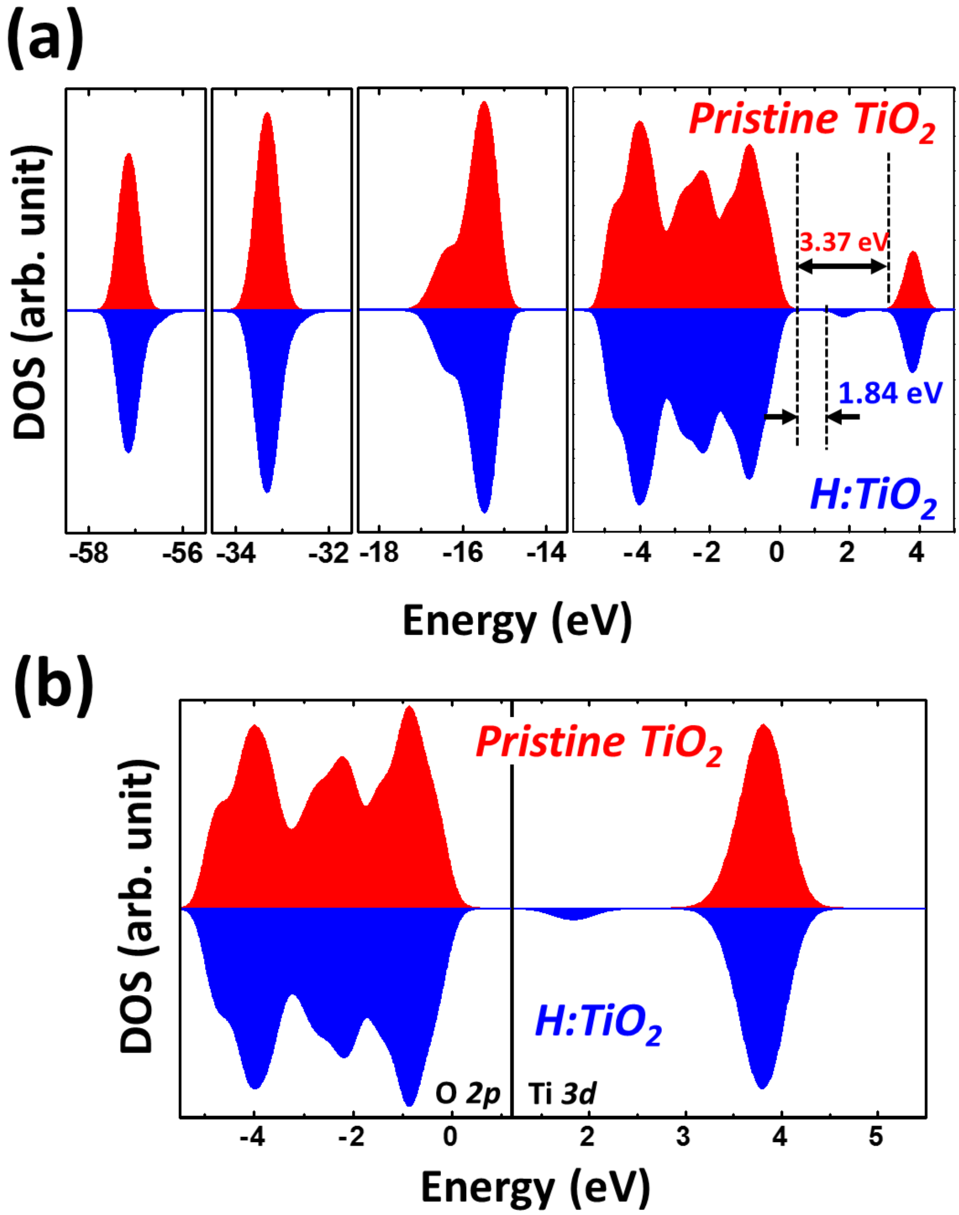


**Figure S7. (a)** Density of states of pristine $TiO_2$ and H:$TiO_2$. Mid-states generated by surface defects can be observed in H:$TiO_2$ with energy level slightly surpass valence band. These states are more remarkable in H:$TiO_2$ and the narrowing band gap can also be observed due to the generation of surface defects. **(b)** Density of states of O *2p* orbital and Ti 3*d* orbital for pristine $TiO_2$ and H:$TiO_2$, respectively.
